# Supplementary material for: The Autonomic Nervous System (ANS)-Immune Network in People Living With HIV
Source: bioRxiv. 2024 Oct 16:2024.10.15.618447. Preprint. [Version 1] doi: 10.1101/2024.10.15.618447 (PMC11507734; doi:10.1101/2024.10.15.618447)
Supplement: Supplementary file 1 [file NIHPP2024.10.15.618447v1-supplement-1.pdf]

Supplemental Table 1: Normalized protein expression levels in immunotypes 1-4, with pairwise comparisons corrected for multiple comparisons.

**Analyte**	**Group 1 median**	**Group 2 median**	**Group 3 median**	**Group 4 median**	***Group 1 versus 2 p-value***	***Group 1 versus 3 p-value***	***Group 1 versus 4 p-value***	***Group 2 versus 3 p-value***	***Group 2 versus 4 p-value***	***Group 3 versus 4 p-value***
4E-BP1	5.174	5.881	5.439	5.362	0.001	0.633	0.169	0.017	0.100	0.824
ADA	5.289	5.749	5.626	4.967	0.035	0.626	0.272	0.254	0.000	0.272
ARTN	-0.266	-0.150	-0.376	-0.281	0.489	0.203	0.929	0.068	0.587	0.576
AXIN1	5.407	6.405	5.869	5.151	0.000	1.000	0.907	0.028	0.000	0.272
Beta-NGF	1.483	1.610	1.498	1.501	0.012	0.230	0.828	0.050	0.029	0.875
CASP-8	1.899	2.926	2.334	1.904	0.000	0.023	0.814	0.017	0.000	0.287
CCL11	7.518	7.162	6.948	7.363	0.164	0.203	0.760	0.071	0.399	0.110
CCL19	9.728	9.701	9.210	8.866	0.703	0.015	0.019	0.095	0.001	0.272
CCL20	7.251	7.361	6.842	6.626	0.908	0.761	0.061	0.016	0.040	0.834
CCL23	11.248	11.195	10.483	11.117	0.937	0.626	0.246	0.001	0.261	0.110
CCL25	5.643	5.440	5.525	4.867	0.787	0.171	0.134	1.000	0.083	0.302
CCL28	1.403	1.433	1.286	1.059	0.316	1.000	0.033	0.136	0.004	0.188
CCL3	5.139	5.234	4.759	4.656	0.937	0.954	0.017	0.088	0.002	0.531
CCL4	5.327	6.009	5.544	5.058	0.082	0.672	0.336	0.056	0.005	0.272
CD244	4.871	5.293	4.843	4.437	0.000	0.015	0.033	0.008	0.000	0.248
CD40	11.058	11.996	11.313	10.148	0.001	0.002	0.023	0.010	0.000	0.015
CD5	5.061	5.142	4.572	4.424	0.671	0.046	0.033	0.002	0.001	0.875
CD6	4.203	4.250	3.288	3.533	0.928	0.388	0.058	0.000	0.011	0.248
CD8A	8.894	9.014	8.185	8.396	0.960	0.271	0.099	0.016	0.027	0.875
CDCP1	3.122	2.605	2.512	2.084	0.042	0.271	0.004	0.814	0.080	0.165
CSF-1	8.762	8.708	8.431	8.264	0.935	0.203	0.044	0.017	0.003	0.481
CST5	6.679	6.937	7.337	6.183	0.563	0.008	0.044	0.299	0.001	0.005
CX3CL1	3.497	3.203	3.174	2.971	0.339	0.020	0.017	0.969	0.105	0.260
CXCL1	8.666	9.664	9.147	8.135	0.000	0.145	0.294	0.164	0.000	0.110
CXCL10	8.328	7.882	7.250	7.367	0.069	0.969	0.017	0.017	0.083	0.845
CXCL11	8.617	8.865	7.828	9.190	0.794	0.000	0.703	0.000	0.753	0.038
CXCL5	11.072	12.639	12.068	9.645	0.000	0.461	0.087	0.112	0.000	0.022
CXCL6	7.157	8.256	7.109	7.066	0.000	1.000	0.561	0.000	0.000	0.747

Analyte	Group 1 median	Group 2 median	Group 3 median	Group 4 median	*Group 1 versus 2 p-value*	*Group 1 versus 3 p-value*	*Group 1 versus 4 p-value*	*Group 2 versus 3 p-value*	*Group 2 versus 4 p-value*	*Group 3 versus 4 p-value*
CXCL9	7.264	6.712	5.894	6.552	0.056	0.090	0.033	0.010	0.656	0.137
DNER	8.144	8.309	8.291	7.787	0.301	0.090	0.118	0.963	0.006	0.110
EN-RAGE	2.904	3.339	2.677	2.073	0.089	0.775	0.294	0.039	0.000	0.091
FGF-19	7.348	7.631	8.501	7.425	0.870	0.954	0.584	0.008	0.725	0.107
FGF-21	4.770	4.303	3.831	3.377	0.427	0.090	0.033	0.461	0.109	0.545
FGF-23	0.788	0.866	0.741	0.381	0.960	0.848	0.087	0.926	0.056	0.097
FGF-5	0.766	0.855	0.694	0.748	0.526	0.128	0.607	0.315	0.115	0.747
Flt3L	8.465	8.362	8.158	8.091	0.483	0.087	0.158	0.268	0.399	0.865
GDNF	0.729	0.814	0.828	0.492	0.743	0.282	0.306	0.994	0.109	0.356
HGF	7.509	7.518	7.037	6.871	0.937	0.743	0.044	0.023	0.002	0.692
IFN-gamma	7.349	6.890	6.232	6.880	0.483	0.848	0.231	0.050	0.587	0.434
IL-1 alpha	-0.044	0.041	0.173	0.138	0.339	0.667	0.790	0.855	0.602	0.692
IL10	1.399	1.997	1.628	1.433	0.038	0.020	0.465	0.091	0.007	0.481
IL-10RA	-0.343	-0.327	-0.576	-0.494	0.928	0.602	0.436	0.645	0.216	0.952
IL-10RB	5.572	5.604	5.451	5.301	0.928	0.102	0.087	0.605	0.038	0.545
IL-12B	4.762	4.916	3.929	4.321	0.563	0.324	0.318	0.000	0.121	0.151
IL13	-0.741	-0.746	-0.709	-0.666	0.728	0.271	0.486	0.933	0.753	0.875
IL-15RA	0.275	0.215	0.060	0.303	0.884	0.103	0.561	0.268	0.740	0.638
IL-17A	-0.225	0.095	-0.519	-0.442	0.301	0.090	0.907	0.010	0.255	0.545
IL-17C	1.498	1.284	1.037	0.675	0.178	0.203	0.071	1.000	0.222	0.287
IL18	9.135	9.021	8.279	8.492	0.935	0.672	0.109	0.019	0.088	0.725
IL-18R1	5.811	5.810	5.490	5.390	0.960	0.923	0.109	0.009	0.023	0.875
IL2	-0.674	-0.567	-0.564	-0.653	0.153	0.672	0.387	0.969	0.915	0.834
IL-20	-0.395	-0.056	-0.269	-0.237	0.006	0.672	0.631	0.046	0.183	0.905
IL-20RA	-0.283	-0.275	-0.401	-0.012	0.519	0.402	0.104	0.616	0.165	0.260
IL-22 RA1	0.235	0.778	0.421	0.724	0.129	0.090	0.387	0.436	0.747	0.607
IL-24	0.638	0.939	0.805	1.025	0.264	0.336	0.436	0.723	0.887	0.875
IL-2RB	-0.250	-0.134	-0.218	-0.308	0.316	0.090	0.766	0.994	0.231	0.545
IL33	-0.016	0.151	0.192	0.119	0.035	0.388	0.401	0.933	0.407	0.545
IL4	-0.640	-0.730	-0.441	-0.974	0.934	0.492	0.766	0.315	0.433	0.173
IL5	-1.003	-0.560	-0.980	-0.869	0.043	0.775	0.465	0.104	0.747	0.670

Analyte	Group 1 median	Group 2 median	Group 3 median	Group 4 median	*Group 1 versus 2 p-value*	*Group 1 versus 3 p-value*	*Group 1 versus 4 p-value*	*Group 2 versus 3 p-value*	*Group 2 versus 4 p-value*	*Group 3 versus 4 p-value*
IL6	2.409	2.000	1.966	1.443	0.316	0.388	0.017	0.497	0.049	0.481
IL7	0.479	1.118	0.771	-0.017	0.001	0.672	0.380	0.039	0.000	0.107
IL8	5.002	5.032	4.619	4.693	0.663	0.002	0.655	0.112	0.261	0.845
LAP TGF-beta-1	5.420	5.779	5.397	4.835	0.001	0.206	0.061	0.010	0.000	0.061
LIF	-1.031	-0.830	-0.928	-0.792	0.025	0.819	0.294	0.436	0.915	0.865
LIF-R	2.364	2.424	2.248	2.077	0.743	0.388	0.017	0.436	0.002	0.097
MCP-1	11.367	11.053	10.390	11.095	0.249	0.775	0.401	0.009	0.671	0.022
MCP-2	8.178	8.938	8.588	7.586	0.072	0.492	0.044	0.497	0.000	0.005
MCP-3	0.752	0.918	0.969	0.561	0.181	0.508	0.306	0.524	0.006	0.151
MCP-4	14.838	15.176	14.601	14.532	0.071	0.775	0.436	0.003	0.007	0.875
MMP-1	13.292	14.241	13.549	12.546	0.042	0.633	0.095	0.058	0.000	0.110
MMP-10	7.809	7.659	8.062	7.176	0.819	0.206	0.044	0.196	0.043	0.028
NRTN	-0.913	-0.807	-0.779	-0.813	0.439	0.619	0.679	0.933	0.952	0.981
NT-3	2.042	2.171	2.201	1.832	0.291	0.848	0.318	0.713	0.049	0.302
OPG	9.245	9.041	9.015	8.598	0.512	0.171	0.058	0.994	0.029	0.272
OSM	2.846	2.814	2.095	2.811	0.960	0.090	0.828	0.039	0.686	0.107
PD-L1	4.967	5.615	4.943	4.816	0.012	0.045	0.087	0.010	0.000	0.531
SCF	8.048	8.144	8.067	7.573	0.877	0.090	0.294	0.969	0.029	0.151
SIRT2	2.974	4.340	3.556	2.288	0.000	0.013	0.465	0.040	0.000	0.048
SLAMF1	1.221	0.999	0.894	0.568	0.012	0.848	0.002	0.845	0.012	0.173
ST1A1	3.993	4.748	3.966	3.399	0.010	0.312	0.104	0.013	0.000	0.097
STAMBP	5.246	6.302	5.852	4.514	0.000	0.383	0.457	0.010	0.000	0.038
TGF-alpha	2.161	2.164	1.925	1.781	0.826	0.271	0.018	0.008	0.001	0.434
TNF	2.672	2.570	2.180	2.275	0.935	0.848	0.099	0.010	0.029	0.952
TNFB	3.522	3.363	3.174	3.275	0.433	0.383	0.231	0.323	0.617	0.834
TNFRSF9	5.229	4.998	4.440	4.983	0.696	0.388	0.272	0.007	0.407	0.272
TNFSF14	3.789	4.430	3.725	3.179	0.003	0.089	0.124	0.010	0.000	0.381
TRAIL	7.670	7.712	7.257	7.559	0.656	0.854	0.387	0.046	0.080	0.845
TRANCE	3.685	4.030	3.368	3.776	0.197	0.108	1.000	0.010	0.222	0.545
TSLP	-0.007	-0.184	0.251	0.157	0.563	0.854	0.766	0.046	0.420	0.545
TWEAK	8.225	8.470	8.086	7.994	0.068	0.667	0.066	0.017	0.000	0.151

Analyte	Group 1 median	Group 2 median	Group 3 median	Group 4 median	*Group 1 versus 2 p-value*	*Group 1 versus 3 p-value*	*Group 1 versus 4 p-value*	*Group 2 versus 3 p-value*	*Group 2 versus 4 p-value*	*Group 3 versus 4 p-value*
uPA	9.811	9.943	9.551	9.583	0.175	0.103	0.261	0.039	0.017	0.952
VEGFA	10.820	10.976	10.616	10.583	0.193	0.122	0.124	0.019	0.006	0.511
